# Factors influencing same-day discharge after minimally invasive hysterectomy for malignant and non-malignant gynecological diseases: a systematic review and meta-analysis

**DOI:** 10.3389/fonc.2023.1307694

**Published:** 2024-01-09

**Authors:** Jia Liu, Yali Chen, Xin Tan, Hengxi Chen

**Affiliations:** ^1^ Pathology Department, West China Second University Hospital, Sichuan University, Chengdu, Sichuan, China; ^2^ Key Laboratory of Birth Defects and Related Diseases of Women and Children (Sichuan University), Ministry of Education, Chengdu, Sichuan, China; ^3^ Gynaecology and Obstetrics, West China Second University Hospital, Sichuan University, Chengdu, Sichuan, China; ^4^ Day Surgery Department, West China Second University Hospital, Sichuan University, Chengdu, Sichuan, China

**Keywords:** same-day discharge, hysterectomy, minimally invasive surgery, systematic review, meta-analysis

## Abstract

**Objective:**

To explore the factors influencing the successful implementation of same-day discharge in patients undergoing minimally invasive hysterectomy for malignant and non-malignant gynecological diseases.

**Method:**

We searched PubMed, Embase, Cochrane Central Register of Controlled Trials, International Clinical Trials Registry Platform, and Clinical Trials.gov from inception to May 23, 2023. We included case-control and cohort studies published in English reporting same-day discharge factors in patients undergoing minimally invasive hysterectomy for malignant and non-malignant gynecological diseases. STATA 16.0 was used for the meta-analysis. Risk factors were assessed using odds ratios (OR) (relative risk (RR)/hazard ratios (HR)) with 95% confidence intervals (CI), and logistic regression determined the same-day discharge rate (%).

**Results:**

We analyzed 29 studies with 218192 patients scheduled for or meeting same-day discharge criteria. The pooled rates were 50% (95% CI 0.46-0.55), and were similar for malignant and non-malignant gynecological diseases (48% and 47%, respectively). In terms of basic characteristics, an increase in age (OR: 1.03; 95% CI: 1.01–1.05), BMI (OR: 1.02; 95% CI: 1.01–1.03), and comorbidities including diabetes and lung disease were risk factors affecting SDD, while previous abdominal surgery history (OR: 1.54; 95% CI: 0.93–2.55) and hypertension (OR: 1.53; 95% CI: 0.80–2.93) appeared not to affect SDD. In terms of surgical characteristics, radical hysterectomy (OR: 3.46; 95% CI: 1.90–6.29), surgery starting after 14:00 (OR: 4.07; 95% CI: 1.36–12.17), longer surgical time (OR: 1.03; 95% CI: 1.01–1.06), intraoperative complications (OR: 4.68; 95% CI: 1.78–12.27), postoperative complications (OR: 3.97; 95% CI: 1.68–9.39), and surgeon preference (OR: 4.47; 95% CI: 2.08–9.60) were identified as risk factors. However, robotic surgery (OR: 0.44; 95% CI: 0.14–1.42) and intraoperative blood loss (OR: 1.16; 95% CI: 0.98–1.38) did not affect same-day discharge.

**Conclusions:**

An increase in age, body mass index, and distance to home; certain comorbidities (e.g., diabetes, lung disease), radical hysterectomy, surgery starting after 14:00, longer surgical time, operative complications, and surgeon preference were risk factors preventing same-day discharge. Same-day discharge rates were similar between malignant and non-malignant gynecological diseases. The surgery start time and body mass index have a greater impact on same-day discharge for malignant diseases than non-malignant diseases.

## 1 Introduction

Same-day discharge (SDD) for patients who have undergone hysterectomy is becoming more common with the advancement in minimally invasive surgery including its application in treating benign diseases and malignant tumors (1–4). After a minimally invasive hysterectomy, overnight hospitalization is common to monitor perioperative complications such as hemorrhage, blood pressure liability, desaturation, possible intraoperative bladder/ureteral or bowel injuries, or for immediate detection in case of postoperative pain. Experts believe perioperative complications should be identified intraoperatively, requiring immediate admission or a few days after discharge (5). Hence, prolonged hospitalization does not change the readmission rate resulting from complications (6).

There have been reports of lower healthcare costs, better utilization of scarce medical resources, and higher patient satisfaction following the successful application of SDD in minimally invasive hysterectomy without compromising outcomes (7). In the United States, annual hysterectomy costs are over $5 billion (8). A retrospective study by Schiavone et al., reported that the cost of a one-day discharge following a laparoscopic hysterectomy was $207 greater than that of patients discharged on the same day (9). In addition, SDD implementation can allow more patients to obtain high-quality medical resources in areas with limited medical resources.

Minimally invasive hysterectomy is a method to treat many benign or malignant gynecological diseases (10–13). Although the safety, feasibility, and economy of implementing SDD in minimally invasive hysterectomy for benign or malignant gynecological diseases have been confirmed in a series of studies, further research is required on patient and surgical factors that affect its application (14, 15). This study explored the factors affecting the successful implementation of SDD in minimally invasive hysterectomies to enhance the promotion and application of SDD.

## 2 Methods

### 2.1 Protocol registration

This meta-analysis was performed according to the Preferred Reporting Items for Systematic Reviews and Meta-analyses (PRISMA) guidelines and was registered with the International Prospective Register of Systematic Reviews (CRD42023425260) (16).

### 2.2 Eligibility criteria

All potentially eligible studies, including case-control and cohort studies, published in English were considered. The inclusion criteria were: (a) evaluation of factors influencing SDD in patients undergoing minimally invasive hysterectomy for malignant and non-malignant gynecological diseases, (b) effect data including odds ratios (OR), relative risk (RR), or hazard ratios (HR) with 95% confidence intervals (CI) provided or calculation of these data enabled, and (c) if data subsets had been published in more than one article, that with the largest sample size was included. The exclusion criteria were as follows: (a) redundant publications, (b) incomplete data, (c) and conference abstracts and reviews.

### 2.3 Search strategy and study selection

We searched PubMed, Embase, Cochrane Central Register of Controlled Trials (CENTRAL), International Clinical Trials Registry Platform, and Clinical Trials.gov from inception to May 23, 2023. The reference lists of published reviews and retrieved articles were checked for additional trials. The search terms were: “hysterectomy,” “same day discharge,” “outpatient surgery,” “influencing factor,” “risk factor,” “related factor,” and “factor.”

Two researchers (HC and LH) independently screened titles and abstracts to assess the eligibility of the studies and independently read the full texts of all potential articles for further evaluation. Disagreements between the authors were resolved through discussion with a third researcher (XT).

### 2.4 Data extraction

Two independent reviewers (JL and YC) extracted data in duplicate and recorded it in a standardized database. We used a predefined extraction form that included the methods, study quality, participants, and outcomes. The authors were blinded to the trial authors, institutions, sources of funding, and acknowledgments. We attempted to acquire missing data by contacting the authors via email; however, no replies were received.

### 2.5 Risk of bias assessment

Two reviewers (JL and YC) independently assessed the quality of the included studies. Differences were resolved by discussion, and if no consensus was reached, a third review author (XT) was involved. Cohort studies included in the prognosis analysis were assessed using the Newcastle–Ottawa Scale (NOS) based on three categories: selected cases, comparability of groups, and assessment of outcomes. Studies awarded six or more stars were classified as high-quality.

### 2.6 Statistical analysis

We conducted the meta-analysis using STATA 16.0 (StataCorp., College Station, TX). We employed ORs (relative risks (RRs)/hazard ratios (HRs)) with 95% CI to combine data assessing risk factors. Only those risk factors investigated in at least two studies were included in the meta-analysis. Statistical significance was set at P <0.05. The SDD rate (%) was determined using logistic regression analysis. The heterogeneity between studies was assessed using the I^2^ test: I^2^ <30% was considered low heterogeneity, I^2^ 30–50% was designated to have moderate heterogeneity, and I^2^ ≥50% was considered high heterogeneity (17). When there was substantial heterogeneity, a random-effects model was used to combine the data. Otherwise, a fixed effects model was used. Publication bias was evaluated using a funnel plot, and statistical assessment was performed using the Egger test (18).

## 3 Results

### 3.1 Study selection and characteristics

**Figure 1:**
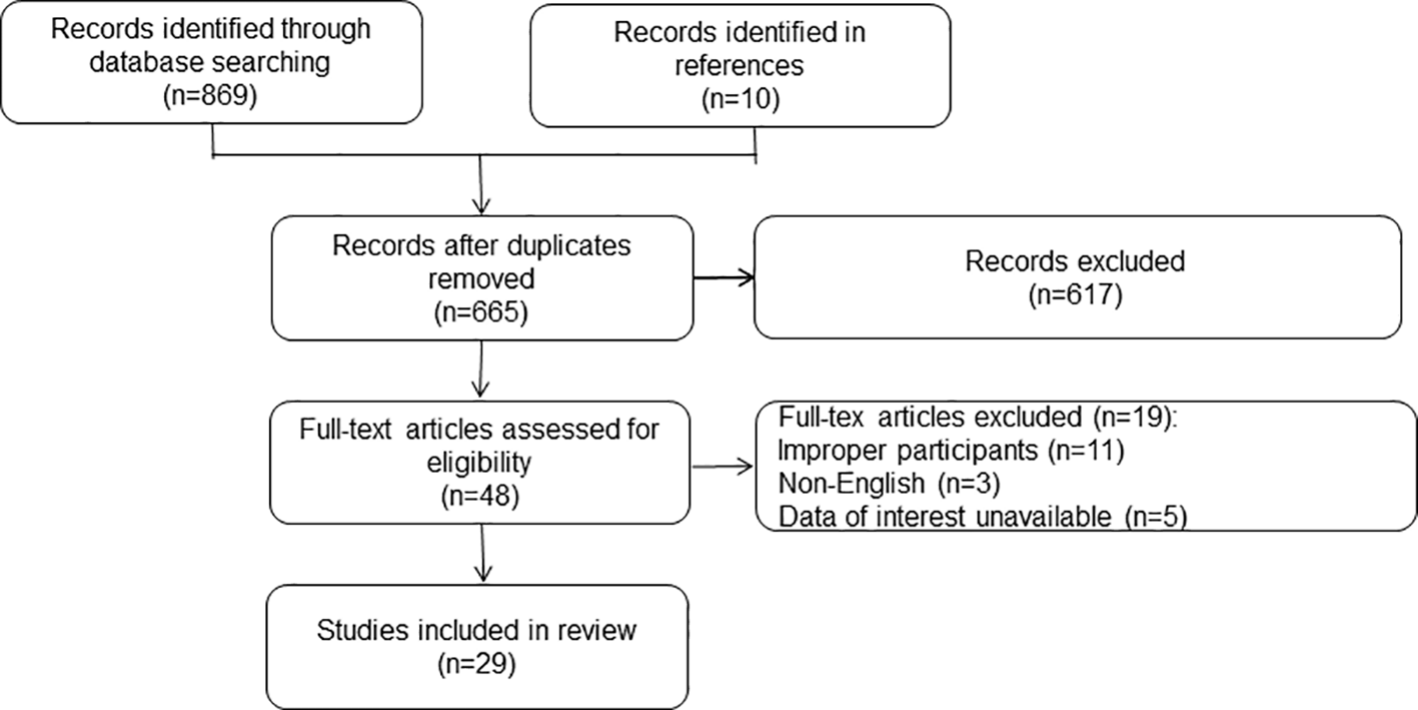
Flowchart of the study selection.

The study selection process is illustrated in **Figure 1**. A total of 665 articles were retrieved after removing duplicates. After screening the titles and abstracts, 48 full texts were retrieved for subsequent assessment. After reading the full texts, 19 articles were excluded. Finally, 29 studies were included with 218,192 patients scheduled for SDD or who met the SDD criteria (5, 7, 8, 19–44). No randomized controlled trials were found. All the included studies were retrospective cohort studies and were awarded six or more stars according to the NOS criteria. The general characteristics of the included studies are summarized in **Table 1**.

**Table 1:** The characteristics of the included studies.

Study	Country	Study type	Age (mean)	BMI (mean)	Hysterectomy	Additional surgical procedures	Total patients (N)	SDD (n)	Indication for surgery
Rivard 2015 (12)	America	Retrospective study	56.7	34	Robotic-assisted MIH	Salpingo-oophorectomy, omentectomy, appendectomy, lymphadenectomy, debulking surgery	140	90	Endometrial hyperplasia, endometrial cancer, other cancer. Adnexal, uterine, or cervical abnormality. Risk reducing surgery.
Zhang 2021 (13)	America	Retrospective study	60.2	37.3	Robotic-assisted MIH	Salpingectomy ± oophorectomy, sentinel lymph node biopsy, lymphadenectomy, lysis of adhesions, cystoscopy	158	132	Endometrial cancer
Wield 2022 (14)	America	Retrospective study	58.1	34.1	MIH (simple or radical)	Salpingectomy ± oophorectomy, sentinel lymph node biopsy, lymphadenectomy, lysis of adhesions, ureterolysis, cystectomy, omental surgery, hernia repair	1124	775	Uterine, cervical, ovarian,metastatic, dual primaries cancer/precancer
Gien 2011 (15)	Canada	Retrospective study	54.4	26.9	MIH	Salpingo-oophorectomy, omentectomy, lymphadenectomy	303	147	Endometrial cancer, cervix cancer, ovarian cancer or pelvic mass, benign/preinvasive disease
Lee 2016 (2)	America	Retrospective study	61	31.4	MIH (simple or radical)	NA	9020	729	Endometrial cancer
Philp 2017 (16)	Canada	Retrospective study	43.8	26	MIH (radical)	Sentinel lymph node biopsy, pelvic and/or paraaortic lymphadenectomy	119	75	Cervical cancer
Penner 2014 (17)	America	Retrospective study	60	26	MIH (simple or radical)	Salpingo-oophorectomy, pelvic and/or paraaortic lymphadenectomy	141	118	Cervical cancer, endometrial cancer
Praise 2019 (18)	America	Retrospective study	NA	NA	MIH	NA	17935	1828	Endometrial cancer
Son 2021 (19)	America	Retrospective study	62.2	34.3	MIH	Salpingo-oophorectomy, sentinel lymph node biopsy or lymphadenectomy	86	66	Endometrial cancer
Giannini 2022 (20)	Italy	Retrospective study	64	31	MIH	Salpingo-oophorectomy, sentinel lymph node biopsy or lymphadenectomy, extensive adhesiolysis, omentectomy, appendectomy, hernia repair, prolapse repair, bladder surgery and positioning of the stent	292	117	Endometrial cancer
Haight 2023 (4)	America	Retrospective study	53	45	MIH	Salpingo-oophorectomy, sentinel lymph node biopsy or lymphadenectomy	374	83	Endometrial cancer, complex atypical hyperplasia, pelvic mass, cancer risk reduction, and an alternative cancer diagnosis
Qi 2021 (21)	America	Retrospective study	NA	NA	MIH	NA	5554	2876	Benign disease
AlAshqar 2022 (22)	America	Retrospective study	NA	NA	MIH	Ovarian/tubal surgery, adhesiolysis, prolapse repair, incontinence repair, cystoscopy/cystourethroscopy	1084	238	Benign disease
Moawad 2018 (23)	America	Retrospective study	43.44	32.04	MIH	Cystoscopy, ovarian cystectomy, lysis of Adhesions, excision of endometriosis, uterosacral suspension, ureterolysis	396	312	Benign disease
Sheyn 2017 (5)	America	Retrospective study	46.5	29.5	MIH	Adnexal surgery, adhesiolysis	9096	3032	Benign disease
Tannus 2022 (24)	America	Retrospective cohort study	46.21	28.9	MIH	Adnexal surgery	890	618	Benign disease
Jennings 2015 (25)	America	Retrospective study	46.4	30	MIH	bilateral salpingo-oophorectomy(BSO)	8890	1855	Gynecological disease
Fountain 2017 (26)	America	Retrospective study	52	33.1	MIH	NA	123	43	Benign and malignant indications
Gale 2018 (27)	Canada	Retrospective cohort study	44.4	29.8	MIH	NA	53	44	Benign disease
Lee2014 (28)	America	Retrospective study	52	26.7	robotic-assisted MIH	BSO with sentinel node mapping, pelvic and/or aortic nodal dissection, appendectomy, or omentectomy	200	157	Endometrial cancer, cervical cancer, ovarian cancer, other cancer, benign disease
McAlarnen 2022 (29)	America	Retrospective study	60	32	robotic-assisted MIH	staging	112	36	Benign and malignant indications
Lassen 2012 (30)	Denmark	Retrospective study	NA	NA	MIH	Bilateral salpingo-oophorectomy	26	23	Benign and preinvasive disease
Nensi 2018 (31)	Canada	Retrospective cohort study	47.9	27.9	MIH	salpingectomy, salpingo-oophorectomy, ovarian cystectomy	256	47	Benign disease
Burdick 2011 (32)	America	Retrospective study	45	28	MIH	lysis of adhesions, dilation and curettage, adnexal procedures, and repair of cystotomy	1015	527	Benign disease
Melamed 2015 (33)	America	Retrospective cohort study	NA	NA	MIH	pelvic lymphadenectomy,para-aortic lymphadenectomy, and omentectomy	696	295	Endometrial cancer
Rettenmaier 2012 (34)	America	Retrospective study	NA	NA	MIH	bilateral salpingo-oophorectomy and bilateral pelvic lymph node dissection	26	23	Endometrial cancer
Schiavone 2012 (35)	America	Retrospective study	NA	NA	MIH	NA	128634	34070	Benign disease
Schiff 2019 (36)	America	Retrospective study	46.1	29.9	MIH	NA	31347	6000	Benign disease
Kim 2022 (37)	Canada	Retrospective study	59	32	MIH	Sentinel node assessment, lymphadenectomy	102	76	Uterine cancer, ovarian cancer, cervical cancer, ovarian neoplasm, other

SDD, same-day discharge; MIH, minimally invasive hysterectomy; NA, not available.

### 3.2 SDD rate

**Figure 2:**
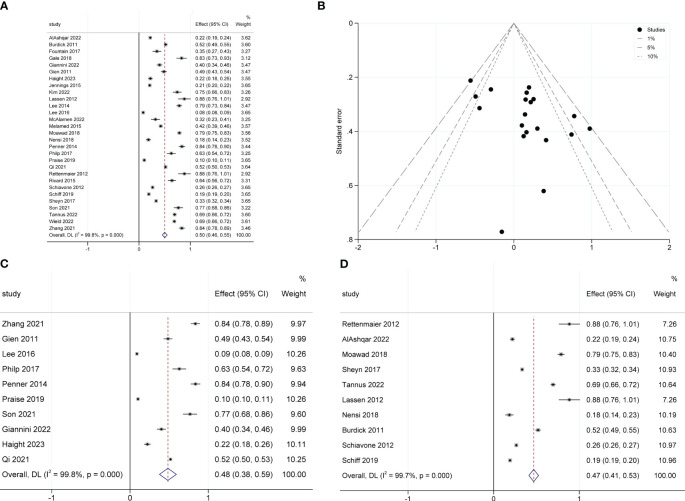
Forest plot of pooled same-day discharge rates **(A)**, funnel plot **(B)**, and the SDD rates for malignant subgroup **(C)** and non-malignant subgroup **(D)**.

The pooled SDD rates were 50% (95% CI 0.46–0.55; I^2^ = 99.8%; 29 studies with 218,192 participants; **Figure 2A**) (5, 7, 8, 19–44). The publication bias in these studies was assessed using a funnel plot (**Figure 2B**), followed by the Egger test (P=0.011), indicating that a high risk of publication bias existed among these studies. Subgroup analysis showed the SDD rates for malignant (OR 48%; 95% CI 0.38-0.59; I^2^ = 99.8%; 10 studies, 172,770 participants; **Figure 2C**) and non-malignant gynecological diseases (OR 47%; 95% CI 0.41-0.53; I^2^ = 99.7%; 10 studies, 33253 participants; **Figure 2D**) were similar.

### 3.3 Factors influencing SDD

#### 3.3.1 Baseline characteristics

**Figure 3:**
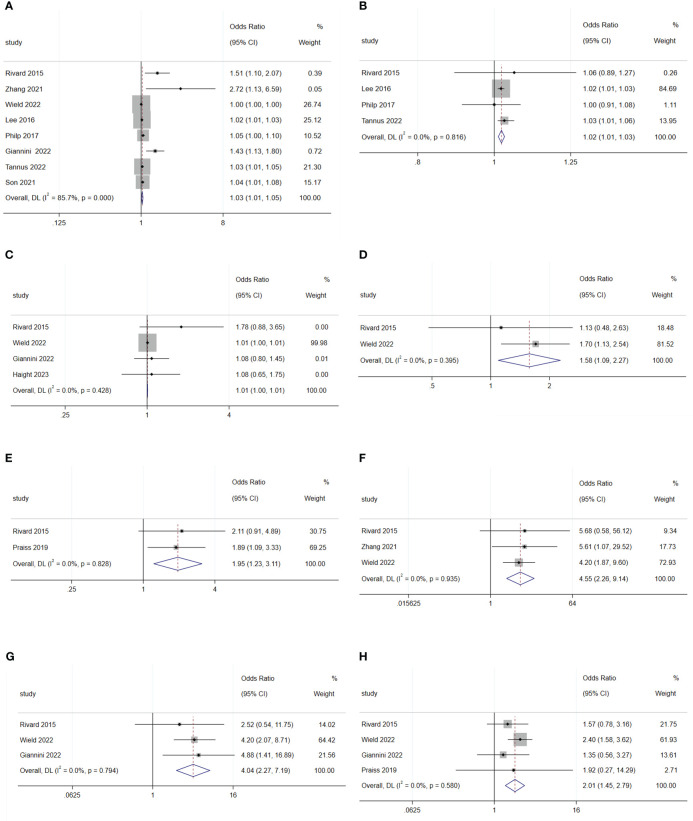
Forest plot of baseline characteristics influencing same-day discharge after minimally invasive hysterectomy in patients: **(A)** age; **(B)** BMI; **(C)** distance to home; **(D)** diabetes; **(E)** lung disease; **(F)** cerebral vascular event; **(G)** deep-vein thrombosis; **(H)** heart disease.

The meta-analysis revealed that an increase in age (OR: 1.03; 95% CI: 1.01–1.05; I^2^ = 85.7%; **Figure 3A**), BMI (OR: 1.02; 95% CI: 1.01–1.03; I^2^ = 0.0%; **Figure 3B**), and distance from home (OR: 1.01; 95% CI: 1.00–1.01; I^2^ = 0.0%; **Figure 3C**) were disadvantageous factors for SDD (5, 7, 19–21, 23, 26, 27, 31).

**Figure 4:**
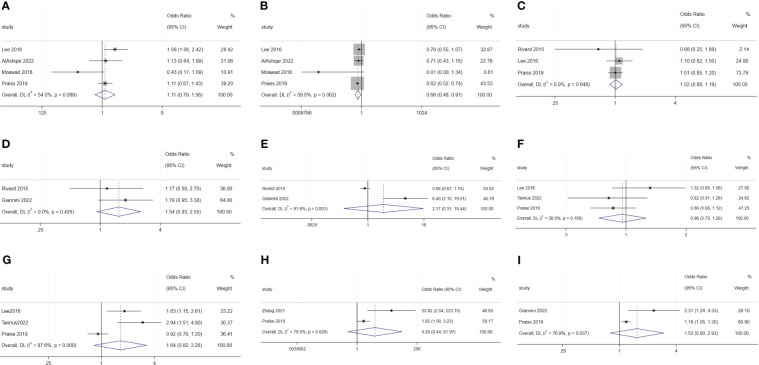
Forest plot of baseline characteristics not influencing same-day discharge after minimally invasive hysterectomy in patients: **(A)** black or white; **(B)** Hispanic and non-Hispanic people; **(C)** smoking; **(D)** previous abdominal surgery history; **(E)** preoperative hemoglobin levels; **(F)** ASA 2; **(G)** ASA 3; **(H)** ASA 4; **(I)** hypertension.

There was no difference in rates of SDD between Black and White patients (OR: 1.11; 95% CI: 0.79–1.56; I^2^ = 54.0%; **Figure 4A**), between Hispanic and non-Hispanic people (OR: 0.66; 95% CI: 048–0.91; I^2^ = 59.0%; **Figure 4B**), and between smoking and non-smoking populations (OR: 1.02; 95% CI: 0.88–1.19; I^2^ = 0.0%; **Figure 4C**) (5, 19, 25, 29, 30).

Previous abdominal surgery (OR: 1.54; 95% CI: 0.93–2.55; I^2^ = 0.0%; **Figure 4D**) and preoperative hemoglobin levels (OR: 2.17; 95% CI: 0.31–15.44; I^2^ = 91.6%; **Figure 4E**) did not affect SDD (19, 27).

Diabetes (OR: 1.58; 95% CI: 1.09–2.27; I^2^ = 0.0%; **Figure 3D**), lung disease (OR: 1.95; 95% CI: 1.23–3.11; I^2^ = 0.0%; **Figure 3E**), cerebral vascular events (OR: 4.55; 95% CI: 2.26–9.14; I^2^ = 0.0%; **Figure 3F**), deep-vein thrombosis (OR: 4.04; 95% CI: 2.27–7.19; I^2^ = 0.0%; **Figure 3G**), and heart disease (OR: 2.01; 95% CI: 1.45–2.79; I^2^ = 0.0%; **Figure 3H**) were disadvantageous factors for SDD (16, 18, 22, 24). Moreover, different American Society of Anesthesiologists physical status classification system (ASA) status, and hypertension were not predictive factors for SDD (**Figure 4F-I**) (5, 25, 31).

#### 3.3.2 Surgical characteristics

**Figure 5:**
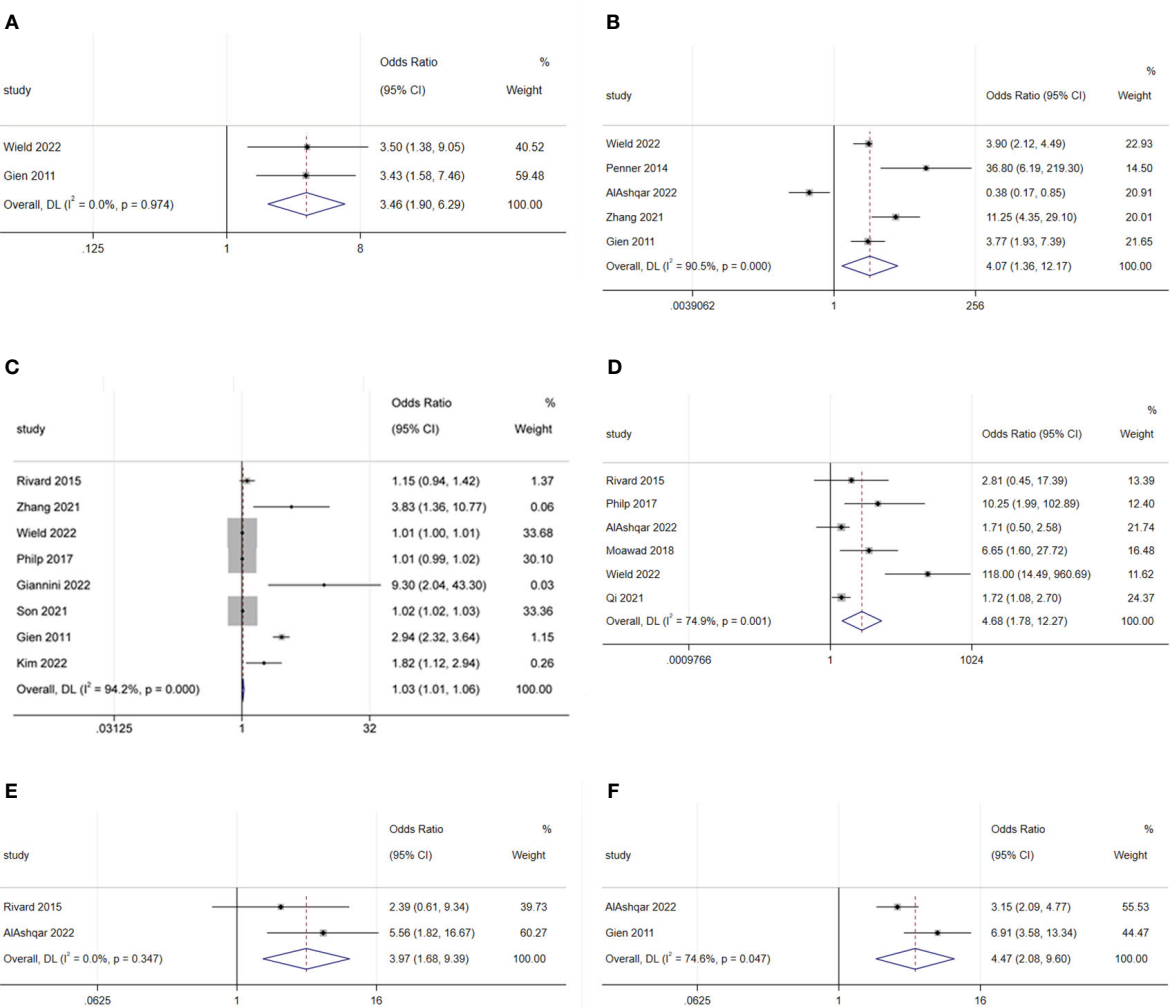
Forest plot of surgical characteristics influencing same-day discharge after minimally invasive hysterectomy in patients: **(A)** radical hysterectomy; **(B)** start of surgery after 14:00; **(C)** increase in surgical time; **(D)** intraoperative complications; **(E)** postoperative complications; **(F)** preferences of surgeons.

**Figure 6:**
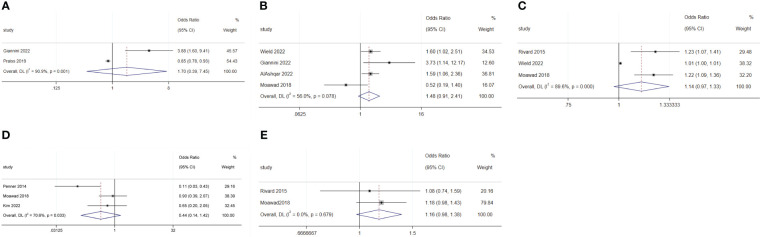
Forest plot of surgical characteristics not influencing same-day discharge after minimally invasive hysterectomy in patients: **(A)** lymphadenectomy; **(B)** adhesiolysis; **(C)** end time of surgery; **(D)** robotic surgery; **(E)** intraoperative blood loss.

The meta-analysis showed that radical hysterectomy (OR: 3.46; 95% CI: 1.90–6.29; I^2^ = 0.0%; **Figure 5A**) was disadvantageous for SDD, while lymphadenectomy (OR: 1.70; 95% CI: 0.39–7.45; I^2^ = 90.9%; **Figure 6A**) and adhesiolysis (OR: 1.48; 95% CI: 0.91–2.41; I^2^ = 56.0%; **Figure 6B**) did not affect SDD (21, 22, 25, 27, 29, 30).

Surgery starting after 14:00 (OR: 4.07; 95% CI: 1.36–12.17; I^2^ = 90.5%; **Figure 5B**) and longer surgical time (OR: 1.03; 95% CI: 1.01–1.06; I^2^ = 94.2%; **Figure 5C**) were disadvantageous factors for SDD (19–24, 26, 27, 29, 44). The end time of surgery did not affect SDD (OR: 1.14; 95% CI: 0.97–1.33; I^2^ = 89.6%; **Figure 6C**) (19, 21, 30).

Robotic surgery had no impact on SDD (OR: 0.44; 95% CI: 0.14–1.42; I^2^ = 70.6%; **Figure 6D**) compared to conventional minimally invasive hysterectomy (24, 30, 44). Intraoperative blood loss (OR: 1.16; 95% CI: 0.98–1.38; I^2^ = 0.00%; **Figure 6E**) also did not affect SDD (19, 30).

Intraoperative complications (OR: 4.68; 95% CI: 1.78 to 12.27; I^2^ = 74.9%; **Figure 5D**) and postoperative complications (OR: 3.97; 95% CI: 1.68 to 9.39; I^2^ = 0.0%; **Figure 5E**) were disadvantageous factors for SDD (19, 21, 23, 28–30). Surgeon preference was an important influencing factor for SDD (OR: 4.47; 95% CI: 2.08–9.60; I^2^ = 74.6%; **Figure 5F**) (22, 29).

#### 3.3.3 Subgroup analysis: influencing factors for malignant diseases and non-malignant diseases

**Figure 7:**
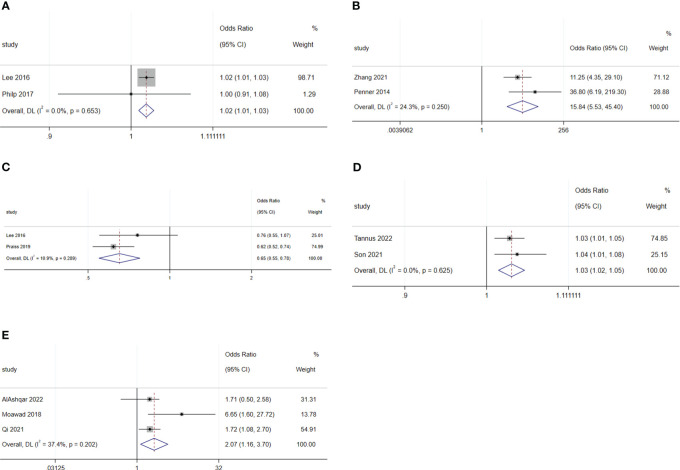
Forest plot of factors influencing same-day discharge after minimally invasive hysterectomy in subgroups: **(A)** (BMI), **(B)** (start time of surgery after 14:00) and **(C)** (Hispanic and non-Hispanic people) for malignant diseases; **(D)** (age) and **(E)** (intraoperative complication) for non-malignant diseases.

**Figure 8:**
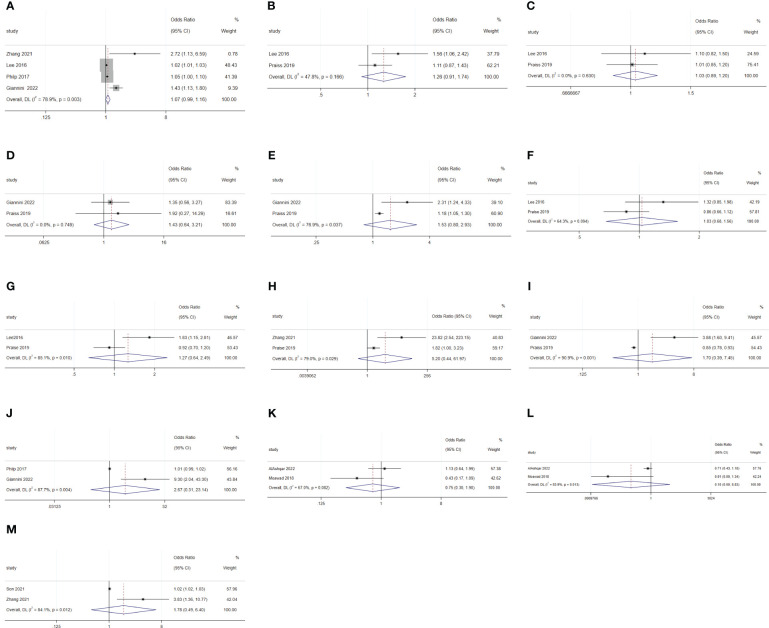
Forest plot of factors not influencing same-day discharge after minimally invasive hysterectomy in subgroups: **(A)** (age), **(B)** (black or white), **(C)** (smoking), **(D)** (heart disease), **(E)** (hypertension), **(F)** (ASA 2), G (ASA 3), **(H)** (ASA 4); **(I)** (lymphadenectomy) and **(J)** (length of surgery) for malignant diseases; **(K)** (black or white), **(L)** (adhesiolysis) and **(M)** (length of surgery) for non-malignant diseases.

For malignant diseases, BMI (OR: 1.02; 95% CI: 1.01–1.02; I^2^ = 0.0%; **Figure 7A**) and surgery starting after 14:00 (OR: 15.84; 95% CI: 5.53–45.4; I^2^ = 24.3%; **Figure 7B**) were disadvantage factors for SDD. Hispanic people were more likely to leave hospital on the same day of surgery (OR: 0.65; 95% CI: 0.55–0.78; I^2^ = 10.9%; **Figure 7C**) (5, 20, 23–25). Age (OR: 1.07; 95% CI: 0.99–1.16; I^2^ = 78.9%; **Figure 8A**), race (Black or White) (OR: 1.26; 95% CI: 0.91 to 1.74; I^2^ = 47.8%; **Figure 8B**), smoking (OR: 1.03; 95% CI: 0.89–1.20; I^2^ = 0.0%; **Figure 8C**), heart disease (OR: 1.43; 95% CI: 0.64–3.21; I^2^ = 0.0%; **Figure 8D**), hypertension (OR: 1.53; 95% CI: 0.80–2.93; I^2^ = 76.9%; **Figure 8E**), ASA 2 (OR: 1.03; 95% CI: 0.68–1.56; I^2^ = 64.3%; **Figure 8F**), ASA 3 (OR: 1.27; 95% CI: 0.64–2.49; I^2^ = 85.1%; **Figure 8G**), ASA 4 (OR: 5.20; 95% CI: 0.44–61.97; I^2^ = 79.0%; **Figure 8H**), lymphadenectomy (OR: 1.70; 95% CI: 0.39–7.45; I^2^ = 90.9%; **Figure 8I**), and length of surgery (OR: 2.67; 95% CI: 0.31–23.14; I^2^ = 87.7%; **Figure 8J**) did not affect SDD.

For non-malignant diseases, age (OR: 1.03; 95% CI: 1.02–1.05; I^2^ = 0.0%; **Figure 7D**) and intraoperative complications (OR: 2.07; 95% CI: 1.16–3.70; I^2^ = 37.4%; **Figure 7E**) were disadvantageous factors for SDD (26, 28–31). Race (Black or White) (OR: 0.75; 95% CI: 0.30–1.90; I^2^ = 67.0%; **Figure 8K**), adhesiolysis (OR: 0.10; 95% CI: 0.00–8.83; I^2^ = 83.9%; **Figure 8L**), and length of surgery (OR: 1.78; 95% CI: 0.49–6.40; I^2^ = 84.1%; **Figure 8M**) did not affect SDD.

## 4 Discussion

We comprehensively reviewed the currently available literature on the risk factors influencing SDD after minimally invasive hysterectomy in patients with malignant and non-malignant gynecological diseases. The pooled SDD rates were 49%, similar to the gynecological malignant and non-malignant diseases. The main risk factors influencing SDD were an increase in age, BMI, and distance to home; certain comorbidities (e.g., diabetes, lung disease, cerebral vascular event, and deep-vein thrombosis); radical hysterectomy; surgery starting after 14:00; longer surgical time; intraoperative complications; postoperative complications; and surgeon preference. However, factors such as previous abdominal surgery history, hypertension, ASA status, robotic surgery, and intraoperative blood loss did not appear to impact SDD. The surgical procedures for gynecological malignancies are usually more complex than the non-malignant diseases; hence, the start time of the surgery and BMI had a greater impact on SDD in these cases.

Since the first report of SDD after a minimally invasive hysterectomy in 1993, despite studies demonstrating the safety and efficacy of SDD in minimally invasive hysterectomy, the SDD rate has not been widely adopted (4, 32). Our study revealed a pooled SDD rate of 50%. A high publication bias existed between the included studies, and the reasons are multifactorial. First, the publication years of the included studies ranged from 2011 to 2022. A growing body of literature has reported increasing rates of SDD over the years (26, 40, 45). Giannini et al. reported that the SDD rate for minimally invasive hysterectomies increased from 13.8% to 88% between 2012 and 2021 (27). Second, there is a paucity of standard patient protocols for SDD in minimally invasive hysterectomies. Patient demographics and preoperative, perioperative, and postoperative characteristics were associated with different SDD rates. Third, the acceptance by doctors and patients varies across regions and medical institutions.

Our review of available evidence indicated that patient demographic variables, such as an increase in age, BMI, and distance to home; comorbidities including diabetes; lung disease; cerebrovascular events; deep-vein thrombosis; and heart disease were also disadvantageous factors for SDD (45). Rivard et al. revealed that an age gap of 10 years increases the admission rate by 50% (19). Praise et al., Matern et al., and Rivard et al. reported that patients aged 80-, 75-, and 70-years respectively, are at an increased risk of admission (19, 25, 46). This may provide a cutoff age when patients with SDD are included. Similarly, the rate of SDD decreases with increasing BMI, with rates of 16.3%, 13.7%, and 11.0% among normal-weight, overweight, and obese women, respectively (16). However, another study reported that BMI >40 kg/m (2) did not increase the admission rate. The study illustrated this by introducing robotic surgery, which reduced the conversion rate in obese patients (47, 48). Thus, in the future, BMI may not be a contraindication for SDD in minimally invasive surgery (49). A cohort of studies revealed that comorbidities are unfavorable factors for SDD in minimally invasive hysterectomy (5, 6, 26, 31). Ji et al. and Lee et al. reported that the admitted group had older age, higher BMI, and more comorbidities associated with more complex surgical procedures that affected the SDD rate (5, 26). Our meta-analysis revealed that distance to family is a negative factor for SDD. Patients may refuse SDD because of concerns regarding postoperative complications and inconvenient readmission. However, the effect of distance to family remains controversial.^7.19,21,27^ A low 30 day-readmission rate and preoperative consultations for SDD surgery may weaken the impact of family distance. A meta-analysis including 16423 patients who underwent minimally invasive surgery and SDD by a gynecological oncologist showed no statistically significant differences in complications and readmission rates within 30 days after surgery when compared with those in patients who underwent SDD.

The surgical risk factors for SDD in minimally invasive hysterectomy include preoperative, intraoperative, and postoperative variables. In our meta-analysis, radical hysterectomy, surgery starting after 14:00, increased surgical time, and postoperative/intraoperative complications negatively affected SDD. One study found that the risk of hospitalization increased for every 30-minute increase in surgical time and every 1-hour delay in surgical completion time (16). This can be used as a guide for the surgery starting time and serve as a reference for the cutoff point of SDD. For radical hysterectomy with a longer surgical time, surgeons should provide sufficient preoperative consultation to patients (19, 50).

Surgeon preference is an important factor in SDD. Although it has been reported that the rehospitalization rate and incidence of postoperative complications after minimally invasive hysterectomy with SDD are very low, SDD is not implemented in 38.3% of patients because of doctors concerns about patient safety (27). This reminds us that establishing standardized inclusion procedures, ensuring the smoothness of postoperative readmission channels, and sufficient doctor-patient communication may reduce surgeons’ anxiety and improve the rate of SDD implementation.

Our meta-analysis found that the SDD rates for malignant and non-malignant gynecological diseases are similar, which will enhance the confidence of doctors and patients in SDD for malignant gynecological diseases. The surgical procedures for gynecological malignancies are usually more complex than those for benign diseases; hence, the start time of the surgery and BMI of the patient have greater impacts on SDD.

We followed a review protocol for the study selection, data extraction, and analysis. Two review authors independently performed study selection, data extraction, and assessment of the risk of bias. Standardized data extraction forms were used in this study. However, this study has some limitations. First, all the included studies were retrospective which have intrinsic restrictions. Second, a random-effects model was used for most analyses. The limitations of this approach were the down-weighting of large studies when statistical heterogeneity was present and assigning equal weighting to the combined studies.

An increase in age, BMI, distance to home, certain comorbidities (including diabetes, lung disease, cerebral vascular event, deep-vein thrombosis, and heart disease), radical hysterectomy, surgery starting after 14:00, longer surgical time, operative complications, and surgeon preference were risk factors preventing SDD. In contrast, previous abdominal surgery, hypertension, ASA status, robotic surgery, and intraoperative blood loss do not appear to affect SDD. The SDD rates of malignant and non-malignant gynecological diseases are similar, which will enhance the confidence of doctors and patients on the day of discharge after surgery for malignant gynecological diseases. Compared to non-malignant diseases, the start time and BMI have a greater impact on SDD for malignant diseases.

In conclusion, sufficient preoperative consultation, skilled surgeons’ participation, early surgical times, and avoidance of complications are beneficial for the successful implementation of SDD. Notably, gynecological malignancies are not a risk factor affecting successful SDD, but relatively complex surgeries should begin before 14:00. Adequate operative support can reduce patients’ and surgeons’ concerns about the safety of SDD and improve its successful application.

## Author contributions

JL: Conceptualization, Data curation, Methodology, Writing – original draft. YC: Data curation, Writing – review & editing. XT: Writing – review & editing. HC: Methodology, Writing – original draft, Writing – review & editing.
